# Criminal Abortion

**Published:** 1887-07

**Authors:** 


### CRIMINAL ABORTION.

It is unpleasant to call attention so frequently to this disgusting crime, but the cumulative evidence of its increase and the difficulties of its detection and punishment imperatively demand that it be done. The newspapers throughout the country teem with notices of its commission, brought to light by the accidental death of an adult victim, or the discovery of a ghastly body of a murdered infant.

The comparative safety and secrecy with which an expert scoundrel can commit the crime render discovery difficult and prove that only a few of the many instances of its commission are thrust upon public attention.

When the activity of a coroner, or the enterprise of neighboring swine, discloses the putrifying body of a murdered innocent, the evidence of its maternity is often vague and uncertain, and such testimony as will fix crime upon its perpetrators is impossible.

When the mother becomes the victim, and her sudden death startles the community, a coroner's inquest sometimes demonstrates its cause, but even in these cases, from the secrecy with which the crime may be committed, it is so difficult to trace it to the criminal that convictions are rare and prosecutions difficult and distateful.

To this laxity in the administration of the law is due in part the fearful increase of this offense, which has reached such proportions as in some sections of the United States to seriously affect the annual increase of the population. The census tables show that in many places the families of six to fourteen children which ornamented the decimal returns of the olden time have disappeared, or are found chiefly amongst those of foreign parentage.

If punishment invariably followed the criminal, crime would diminish. But the real cause of the growth of criminal abortion in the United States is to be sought in the relaxation of the obligation of Christian morality in the medical profession and in the hearts and consciences of the women of the land.

Law and religion declare the killing of a child before or after birth as murder. No sophistry can satisfy a Christian conscience that it is anything else. It is only by throwing off all sense of the duty of obedience to the laws of God and man that so inexpressibly hideous a crime can be perpetrated.

And yet it is notorious that in every city medical demons of both sexes more or less openly *out-Herod Herod* in their diabolical practices ; and still, more strange and melancholy is the fact that they find a clientage from whom they extort their wages for the murder of innocence.

This clientage does not come alone from the misguided children of poverty, whom suffering has driven from the path of virtue and fear of disgrace has hurried into crime, but also from the wealthy, the fashionable, the petted children of the church and society whom love of ease and aversion to the cares of maternity induce to defy God's law and rebel against the destiny which he has imposed upon them.

Professional honor, as well as religious training, happily restrain the abler and better members of the medical profession from the commission of this crime, and enable them in proper terms to denounce it, and to resist the importunities and tears of misguided patrons of wealth and respectability who solicit their intervention. The practice of the loathsome outrage is relegated to the degraded camp-followers of a high and honorable calling upon whom argument and denunciation are alike wasted, and whom nothing but the sure vengeance of the law can restrain.

The deep moral depravity of a woman who can think for one moment of consenting to the murder of her unborn offspring is a degradation so profound as to be reached only by lessons of Christianity imparted by its duly-constituted ministers.

The power of this agency cannot be doubted; it is demonstrated in the lessening or prevention of every evil against which it is boldly and energetically directed. It would be easy to furnish statistics showing the success of particular churches which have persistently combatted this evil, as compared with others which have ignored its existence ; but the comparison might seem invidious.

Recognizing, however, the prevalence and growth of this enormous, *beastly* (we beg pardon of the beasts), criminal practice, and the power of religion alone to correct or eradicate it, we earnestly invoke the aid of her ministers of all denominations in the accomplishment of the object.

